# Effects of dietary L-leucine supplementation on testicular development and semen quality in boars

**DOI:** 10.3389/fvets.2022.904653

**Published:** 2022-07-15

**Authors:** Yan Lin, Jiayi Li, Ke Wang, Zhengfeng Fang, Lianqiang Che, Shengyu Xu, Bin Feng, Yong Zhuo, Jian Li, De Wu

**Affiliations:** Key Laboratory for Animal Disease-Resistance Nutrition of the Ministry of Agriculture, Animal Nutrition Institute, Sichuan Agricultural University, Yaan, China

**Keywords:** leucine, boar, testicular development, semen quality, mTOR

## Abstract

Sperm and seminal plasma are rich in leucine, and leucine can promote the protein synthesis. This property makes it an interesting amino acid to increase sperm quality of human and livestock spermatogenesis. The goal of this study was to explore the effects of dietary leucine supplementation on testicular development and semen quality in boars from weaning to 10 months of age. 30 pure-bred, weaned Duroc boars (8.0 ± 1.0 kg) were randomly divided into two groups: control group (CON; fed the basal diet) and leucine group (LEU; fed the basal diet supplemented with 1.2% leucine); then, their body weight and testicular volume were recorded every 4 weeks. Testes were collected for histological and genes expression analysis from 150-day-old boars. Semen was collected and analyzed. Amino acids contents of blood plasma, seminal plasma, sperm, and testes were determined. Dietary supplementation with leucine increased the testicular volume and weight of boars, compared with CON. Sperm viability, sperm count per ejaculation, and average curve speed of sperm in leucine-supplemented boars were increased. Furthermore, leucine supplementation increased the blood plasma and seminal plasma leucine concentrations, and enhanced the gene expressions of branch chain amino acid transaminase, protein kinase B, mammalian target of rapamycin (*mTOR*), and *cyclinb1* in the testes. Interestingly, the expressions of the p-mTOR and mTOR proteins in the testes were also upregulated. Thus, dietary leucine supplementation increased leucine absorption and utilization in the testes, promoted testicular development, and improved semen quality of boars, partly through the mTOR signaling pathway.

## Introduction

Leucine (Leu) is an important branched-chain amino acid (AA). Supplementation of branched-chain AA in a low protein diet in weaned piglets can enhance growth performance, intestinal development, and expression of intestinal AA transporters in piglets (1). Yin et al. found that 21-day-old piglets fed a low-protein diet with 0.55% L-Leu showed increased protein synthesis in skeletal muscles, liver, and other organs, improved average daily weight gain by 61%, and increased phosphorylation levels of mTOR in liver compared with the control group when they were 35 days old (2). Meanwhile, supplementation with 1.19% Leu in low protein diet activated mTOR signaling in longissimus dorsi muscle of piglets, thereby increasing protein synthesis (3).

In addition to the regulation of protein and lipid metabolism, a growing number of studies have shown that Leu may also play an important role in male reproductive performance. Intraperitoneal injection of Leu improved semen quality in zebrafish (4). Kolahian et al. found that supplementation with Leu-containing water for 4 weeks, Wistar rats with diabetes showed significantly improved the semen quality (5). Zhang et al. analyzed the composition of the sperm of ricefield eel, and found the second most abundant metabolite was Leu (6). Additionally, it is generally accepted that Leu concentration was higher in the testes, sperm and semen of boars (7). These studies suggest that Leu may play an important role in the development of testes and spermatogenesis.

Besides, long-term β-hydroxy-β-methylbutyrate (HMB), one of the metabolites of Leu treatment increased the concentrations of insulin-like growth factor 1 (IGF1) and insulin in rats (8). Xu et al. also found that both Leu and HMB stimulated insulin secretion by pancreatic β cells (9). Insulin and IGF1 have a key role in testicular function, such as maintaining the normal number of Leydig cells, maturation of Leydig cells and steroidogenesis (10, 11). Moreover, β-hydroxy-β-methylglutaryl coenzyme A, metabolic end products of Leu, is the precursor of cholesterol synthesis (12), and cholesterol forms the backbone for testosterone (13), which is essential for male reproductive performance. Studies also showed that Leu could stimulate leptin secretion (14), and leucine supplementation can improve leptin sensitivity (15) and affect leptin receptor expressions *via* the mTOR signaling pathway (16), and that leptin caused the testes to release testosterone, which improved the testicular development (17). By reason of the foregoing, Leu has an important role in male reproductive performance. However, the long-term effects of Leu supplementation on testicular development and spermatogenesis have not been studies previously. Therefore, this study investigated the effects of dietary Leu supplementation on testicular development and semen quality of boars to provide theoretical basis and data support for the role of Leu in male reproductive performance in the future.

## Materials and methods

### Animal treatment and experimental designs

Thirty weaned purebred Duroc boars (8.0 kg ± 1.0 kg) were randomly divided into two groups. The experiment was divided into two stages of 30–150 days and 150–300 days. Basal diet formulations of boars at different weight stages were formulated according to the nutrient requirements suggested by the National Research Council (18), and details of the diets are shown in Supplementary Table 1. In the first phase, the CON treatment was fed with the basal diet, and the LEU treatment was fed the basal diet in which 1.2% L-Leu replaced by L-alanine in order to ensure diets were isonitrogenous and isocalory. Leucine was purchased from Huayang Chemical Co., Ltd, Hebei, China. In the second phase, both the LEU and the CON were fed the same diet.

The experiments were carried out at the research farm of Sichuan Agricultural University. Boars were fed *ad libitum* twice a day at 0830 h and 1,430 h and had free access to water before 110 kg body weight (BW). After that, boars were restricted to 2.6 kg/d. Boars were housed individually at an ambient temperature between 18 and 20°C. The feces and urine were cleaned daily, and disinfection and immunization procedures were carried out regularly.

### Detection indicators

#### Growth performance and testicular development

Total amounts of feed (feed intake, surplus, and waste; daily) for each boar, and the BW of each boar (every 4 weeks) were recorded during the study. Average daily gain (ADG), average daily feed intake (ADFI), and feed conversion rate (FCR) were calculated.

The long and short diameters of the testes of each boar were measured using Vernier calipers every 4 weeks. Testicular volume was calculated using formula (19):


$$\begin{array}{l} \text{Testicular volume }({\text{cm}}^{3})\text{ = 0}.\text{5326 }\times \text{ long diameter}\\ \;\times \text{short}\;\text{diameter} \end{array}$$

#### Free amino acids analysis in blood plasma, testis and seminal plasma

On days 1, 57, and 113, blood samples (10 mL) were collected from the anterior vena cava of the boars. Samples were stood at 4°C for 30 min, and then centrifuged at 3,000 rpm for 15 min. The supernatant was divided into 1.5 mL Eppendorf (EP) tubes, and stored at -20°C.

Five 150-day-old boars were randomly selected from each group for anesthetized castration to collect testicular samples. The testes' size and weight were recorded. The samples, 1 cm^3^ (10 mm × 10 mm × 10 mm) in volume, were taken from the left testis and fixed in 10% neutral formalin fixation solution for structure analysis. Then, 1 cm^3^ of the right testicle was placed in a 1.5 mL cryopreservation tube and storage at -80°C until analysis.

After 4 weeks of training, semen was collected weekly for semen quality analysis. The semen sample was filtered into a 10 mL sterile centrifuge tube and centrifuged at 1,000 rpm for 10 min to separate sperm and seminal plasma, and then kept in the - 80°C refrigerator.

The contents of free amino acids (FAA) in boar plasma and seminal plasma were detected by automatic AA analyzer (L-8,900, Hitachi, Tokyo, Japan). The pretreatment of seminal plasma was carried out according to the method of Louis (20). The AA contents of boar testes and sperm were determined using the acid hydrolysis method described in a study by Ren, 2015 (7).

#### Histological analysis of testicular morphology

Testicular tissue sections (5 μm) were made, dyed with hematoxylin and eosin, the internal morphology and structure of testes were observed, and images were retained. In the convoluted seminiferous tubules, the diameter, the number of spermatogonia, and Sertoli cells per tubule, and Seminiferous epithelium height were recorded (21).

#### Sexual desire and semen quality

The boars were trained at the age of 6.5 months and graded by the 5-point scoring system (22). The ejaculation response time and duration of ejaculation of were measured (20).

Semen volume and gel weight were measured in reference to the World Health Organization manual. A computer assisted sperm analysis (CASA) system was used to determine the sperm motility, sperm density, and sperm motility characteristics. Gentian violet staining was used to detected sperm malformation rate. Total spermatozoa and effective spermatozoa per ejaculation were measured in reference to the calculation formula (7). Sperm membrane integrity, sperm acrosome integrity and sperm mitochondrial membrane potential were evaluated by flow cytometry (BD FACSCalibur, Becton, Dickinson and Company, NJ, United States) (23). The determination of sperm membrane integrity and acrosome integrity is using the SYBR-14/PI stains in a Sperm Viability Kit (Molecular Probes L7011, Leiden, The Netherlands) and fluorescein isothiocyanate-peanut agglutinin (FITC-PNA, Sigma) Sperm mitochondrial membrane potentials were detected using JC-1 (lipophilic cation 5,5′,6,6′-tetrachloro-1,1′,3,3′-tetraethylbenzimidazolcarbocyanine iodide), Mitochondrial Membrane Potential Detection Kit (Beyotime Institute of Biotechnology, China).

#### Testicular gene expression

The RNA was extracted from testicular samples by using an RNAsimple Total RNA Extraction Kit (Tiangen, Beijing, China). The cDNA was synthesized according to the commercial reverse transcription kit (Takara BioInc., Japan). The gene expression was determined using a fluorescence, quantitative PCR instrument 7,900 HT (ABI, United States). The internal reference gene was β*-actin*, and the relative expression of mRNA of each gene was calculated using the 2^−ΔΔCT^ method (24).

#### Testicular protein expression

The expression of the p-mTOR, mTOR and P70S6K1 proteins in the testicular tissue of 150-day-old boars was detected by Western Blot (25). Primary antibodies anti-mTOR rabbit monoclonal (ab2732), anti-p70 S6 kinase rabbit polyclonal(ab70271), anti-phospho-mTOR kinase rabbit monoclonal (ab109268) were purchased from Abcam China, whereas the anti-beta-actin monoclonal mouse antibody (dilution 1:2,000; MABT825, Merck Millipore, Germany) was used as a control. Image Lab software was used to analyze the gray value of the strip, β-actin was used as the internal reference, and the relative expression of the target protein was calculated.

#### Statistical analysis

All the data were sorted by Excel 2016 except the routine semen quality and sperm motility characteristics. Morphological section analysis was performed using image-Pro Plus 6.0, and the data were analyzed by *t*-test using SAS9.4 (SAS Inst. Inc., Cary, NC, United States), the results were expressed as mean ± standard deviation (SD). Semen quality analysis and sperm motility characteristics were analyzed by covariance with the time point of semen collection as the covariate. The results were expressed as mean ± standard error of the mean (SEM). The significance level was expressed as *P* < 0.05, and 0.05 < *P* < 0.10 indicated a trend, but when *P* < 0.01, the difference was considered extremely significant.

## Results

### Growth performance

There was no significant difference in initial BW, mean body weight of weeks 4, 8, 12, and 16, ADG, ADFI, and FCR between the CON and LEU (*P* > 0.05) (Table 1).

**Table 1:** Effect of dietary leucine supplementation on growth performance of boars.

**Items**	**Time**	**CON**	**LEU**	***P*** **-value**
Initial weight, kg	1 d	8.10 ± 1.36	7.93 ± 1.12	0.71
ADG, kg/d	1–28 d	0.46 ± 0.09	0.46 ± 0.08	0.97
	29–56 d	0.58 ± 0.10	0.54 ± 0.12	0.29
	57–84 d	0.88 ± 0.14	0.84 ± 0.13	0.37
	85–113 d	1.01 ± 0.09	1.04 ± 0.16	0.62
	1–113 d	0.73 ± 0.09	0.72 ± 0.07	0.60
ADFI, kg/d	1–28 d	0.73 ± 0.01	0.74 ± 0.01	0.82
	29–56 d	1.08 ± 0.14	1.00 ± 0.12	0.13
	57–84 d	1.72 ± 0.25	1.60 ± 0.11	0.14
	85–113 d	2.43 ± 0.11	2.47 ± 0.21	0.53
	1–113 d	1.49 ± 0.11	1.45 ± 0.07	0.22
FCR	1–28 d	1.63 ± 0.22	1.65 ± 0.25	0.79
	29–56 d	1.93 ± 0.40	1.96 ± 0.51	0.90
	57–84 d	1.97 ± 0.29	1.95 ± 0.26	0.82
	85–113 d	2.42 ± 0.26	2.42 ± 0.31	0.98
	1–113 d	2.06 ± 0.24	2.03 ± 0.17	0.77

*CON, control group; LEU, leucine group. ADG, average daily gain; ADFI, average daily feed intake; FCR, feed conversion rate. Values are presented as means ± standard deviation (n = 15)*.

### Testicular development and histological structure

**Figure 1:**
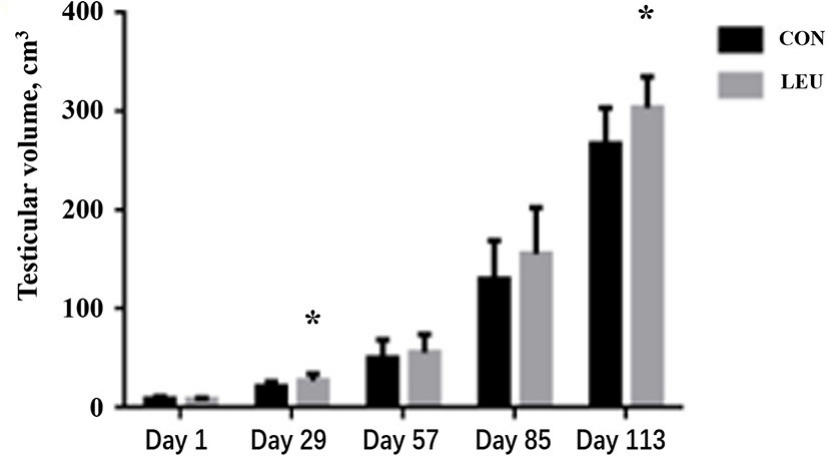
Effect of dietary leucine supplementation on testicular volume in boars. CON, control group; LEU, leucine group. Values are means, with their standard errors represented by vertical bars (*n* = 15), * means *P* < 0.05.

Testicular volume of LEU increased on days 29 and 113(*P* < 0.05), but there was no difference on days 57 and 85, compared to CON (*P* > 0.05) (Figure 1).

The testes weight, and the average length of epididymis of LEU were larger than CON (*P* < 0.05). The total epididymal weight was higher than CON (0.05 < *P* < 0.10), while the testis index of LEU was not different from CON (*P* > 0.05) (Table 2).

**Table 2:** Effects of dietary leucine supplementation on the development of testis and epididymis in 150-days-old boars.

**Items**	**CON**	**LEU**	***P-value***
Testis weight, g	303.5, 41.78	414.6, 26.85	0.02
Testis index, g/kg	3.44, 0.65	4.16, 0.45	0.19
Epididymis weight, g	67.12, 8.50	101.10, 21.73	0.07
Average length of epididymis, cm	15.46, 0.79	20.45, 1.65	0.01

*CON, control group; LEU, leucine group. Values are presented as means ± Standard deviation (n = 15)*.

**Figure 2:**
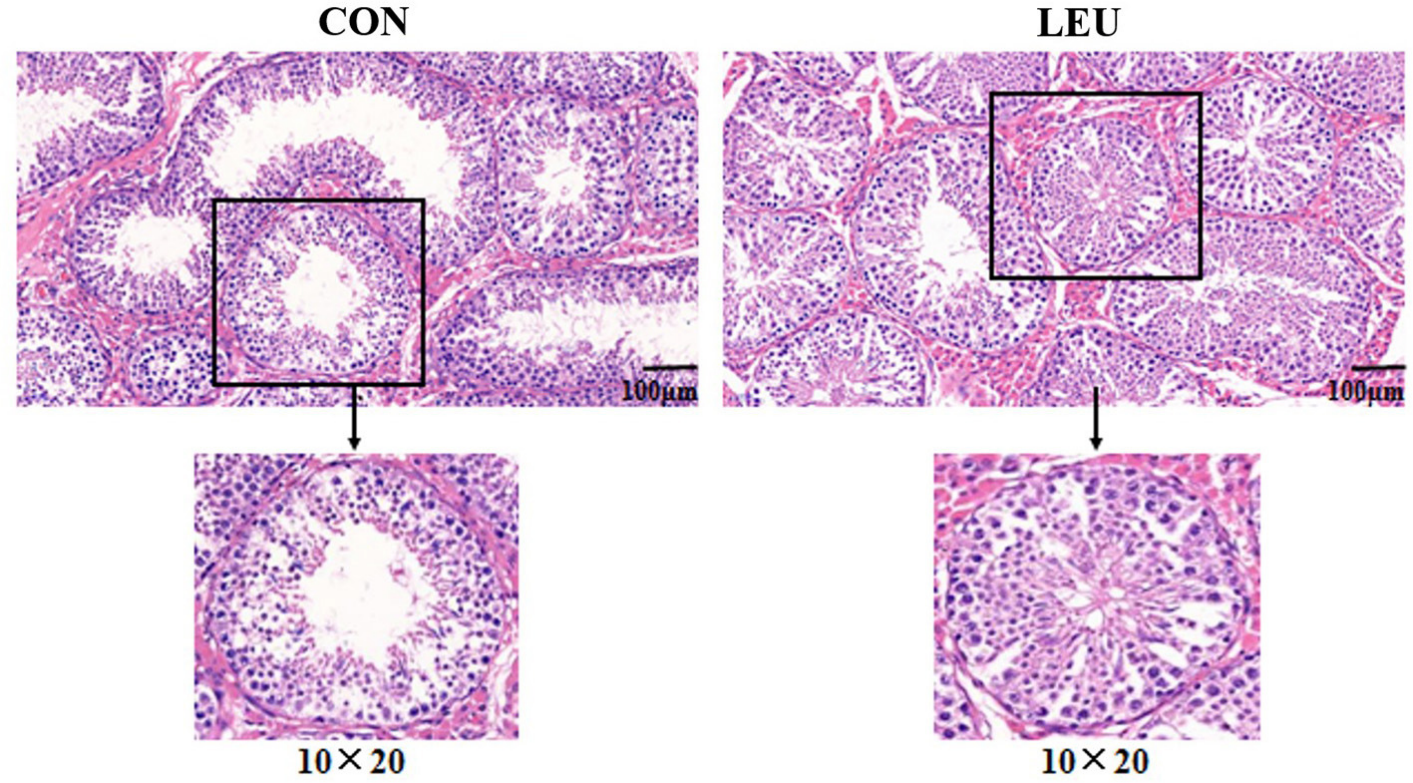
HE staining of 150-days-old testicular tissue in CON and LEU. CON, control group; LEU, leucine group. All the pictures are magnified 200 times.

Boars (150-days-old) in LEU and CON had clear and complete testicular tissue structures and orderly arrangement of various types of cells in the convoluted seminiferous tubules. Compared with CON, the number of layers of cells, and the number of cells in the convoluted seminiferous tubules of the testes were increased in LEU (Figure 2). There is no significant effect on testicular germ cell with the exception of the Sertoli cells, which has a tendency to increase compared to CON (0.05 < *P* < 0.10) (Table 3).

**Table 3:** Effects of dietary leucine supplementation on the histological structure of testis in 150-days-old boars.

**Items**	**CON**	**LEU**	***P-value***
Diameter, μm	206.0, 20.80	215.3, 3.75	0.49
Seminiferous epithelium height, μm	51.67, 5.76	60.07, 4.71	0.12
No. Sertoli cells per tubule	23.17, 0.59	24.04, 24.04	0.07
No. Spermatogonia per tubule	29.24, 3.01	31.07, 0.51	0.36

*CON, control group; LEU, leucine group. Values are presented as means ± SD (n = 15)*.

### Sexual desire and semen quality

The training scores of the LEU group were increased compared to the CON group (*P* < 0.05). There was no difference in the ejaculation response time between LEU and CON (*P* > 0.05), but the duration of ejaculation in LEU was longer than that in CON (*P* < 0.05) (Table 4).

**Table 4:** Effect of dietary leucine supplementation on boar sexual desire.

**Items**	**CON**	**LEU**	***P-value***
Training score	3.41, 0.30	3.96, 0.32	0.03
Ejaculation reaction time, s	169, 18	136, 32	0.11
Duration of ejaculation, s	354, 31	440, 60	0.04

*CON, control group; LEU, leucine group. Values are presented as means ± SD (n = 15)*.

The sperm viability, total sperm count per ejaculation, effective sperm count per ejaculation, and average curve velocity of boars in LEU were increased compared to those in CON (*P* < 0.05). However, there were no differences in semen volume, gel weight, average linear velocity, average path velocity, sperm motility, or sperm deformity rate in LEU compared to CON (*P* > 0.05) (Table 5).

**Table 5:** Effect of dietary leucine supplementation on conventional semen quality of boars.

**Items**	**CON**	**LEU**	***P-value***
Semen volume, mL	92.70, 6.10	102.10, 5.90	0.27
Gel weight, g	27.75, 1.31	28.63, 0.95	0.59
Sperm density, x10^8^ spz/mL	502.71, 15.07	526.67, 23.47	0.40
Sperm viability, %	86.95, 1.66	90.17, 1.33	0.04
Total sperm count per ejaculation, x10^8^ spz	453.88, 22.77	521.60, 20.41	0.03
Effective sperm count per ejaculation, x10^8^ spz	272.83, 12.22	325.54, 18.93	0.02
Average curve velocity, VCL, μm/s	67.14, 4.50	76.99, 2.90	0.03
Average linear velocity, VSL, μm/s	22.04, 1.41	23.21, 0.95	0.43
Average path velocity, VAP, μm/s	31.06, 1.94	33.96, 1.30	0.14
Sperm motility, %	60.85, 2.07	62.04, 2.04	0.67
Sperm abnormality rate, %	6.33, 0.31	6.06, 0.23	0.40

*CON, control group; LEU, leucine group. Values are presented as means ± standard error (n = 15)*.

Compared to CON, the sperm plasma membrane integrity of boars of LEU was higher (0.05 < *P* < 0.10). There was no difference in sperm acrosome integrity or mitochondrial membrane potential (*P* > 0.05) (Table 6).

**Table 6:** Effects of dietary leucine supplementation on plasma membrane integrity, acrosome integrity and mitochondrial membrane potential in boar sperm.

**Items**	**CON**	**LEU**	***P-value***
Sperm plasma membrane integrity rate, %	86.3 ± 7.9	94.6 ± 3.1	0.097
Acrosome integrity rate, %	75.0 ± 7.4	82.2 ± 3.0	0.12
Mitochondrial membrane potential, JC-1^+^%	25.9 ± 9.2	26.9 ± 6.4	0.87

*CON, control group; LEU, leucine group. Values are presented as means ± SD (n = 15)*.

### AA concentration

Initially, there were no significant differences in the contents of FAA in the plasma of each group (*P* > 0.05). On day 57, and 113, the content of free alanine in the plasma of LEU was lower than that in CON (*P* < 0.01). The content of free Leu was significantly higher in LEU than in CON (*P* < 0.05), and the content of free threonine seemed higher on day 57 (0.05 < *P* < 0.10), and was higher on day 113 (*P* < 0.05), compared to CON. There was no difference in the contents of other FAA compared to CON (*P* > 0.05) (Table 7).

**Table 7:** Effect of leucine supplementation on plasma FAA in boars (μmol/L).

**Date**	**Items**	**CON**	**LEU**	***P-value***
1 d	Threonine	98.69, 3.70	89.96, 11.64	0.42
	Alanine	419.20, 11.28	419.60, 27.58	0.98
	Valine	153.10, 13.98	165.70, 12.91	0.32
	Methionine	21.46, 1.20	19.25, 1.48	0.11
	Isoleucine	52.06, 10.33	48.75, 3.57	0.70
	Leucine	84.05, 10.48	75.33, 3.93	0.25
	Phenylalanine	43.31, 5.07	39.91, 5.07	0.46
	Lysine	167.70, 21.41	177.70, 7.33	0.49
	Arginine	73.49, 0.09	64.19, 17.27	0.52
57 d	Threonine	73.76, 6.36	93.30, 33.94	0.09
	Alanine	435.60, 29.20	348.60, 35.39	<0.01
	Valine	144.60, 17.90	124.70, 36.11	0.18
	Methionine	35.39, 5.41	38.58, 3.68	0.23
	Isoleucine	70.19, 6.80	68.14, 13.64	0.69
	Leucine	134.30, 15.11	159.80, 19.02	0.03
	Phenylalanine	83.17, 13.97	90.85, 14.90	0.29
	Lysine	119.70, 22.13	133.80, 20.21	0.20
	Arginine	80.19, 21.83	87.45, 22.91	0.56
113 d	Threonine	93.70, 13.08	113.40, 3.82	0.04
	Alanine	344.30, 18.69	257.20, 14.62	<0.01
	Valine	173.70, 23.95	159.00, 16.32	0.33
	Methionine	40.00, 9.77	38.20, 10.88	0.78
	Isoleucine	61.50, 9.12	58.24, 11.13	0.59
	Leucine	117.20, 19.78	142.80, 9.57	0.04
	Phenylalanine	89.47, 10.19	96.17, 6.97	0.25
	Lysine	138.20, 15.01	147.80, 7.46	0.22
	Arginine	92.50, 22.87	105.80, 14.56	0.34

*CON, control group; LEU, leucine group. Values are presented as means ± Standard deviation (n = 15)*.

The contents of threonine, valine, methionine, isoleucine, phenylalanine, lysine, and alanine in the testes of 150-days-old boars in the LEU group was the same as the CON group (*P* > 0.05), while Leu, aspartic acid, glutamic acid, and total acid hydrolyzed AA showed an increasing trend (0.05 < *P* < 0.10) (Table 8).

**Table 8:** Effect of dietary leucine supplementation on AA composition of testis in 150-days-old boars.

**Items (%)**	**CON**	**LEU**	***P-value***
Aspartic acid	4.75 ± 0.15	5.06 ± 0.02	0.07
Threonine	2.38 ± 0.10	2.45 ± 0.02	0.29
Serine	2.61 ± 0.01	2.63 ± 0.12	0.85
Glutamate	7.84 ± 0.03	8.53 ± 0.34	0.07
Glycine	3.46 ± 0.59	3.53 ± 0.08	0.85
Alanine	3.12 ± 0.18	3.25 ± 0.07	0.29
Cysteine	0.40 ± 0.29	0.66 ± 0.04	0.13
Valine	2.60 ± 0.08	2.69 ± 0.07	0.22
Methionine	0.93 ± 0.15	0.97 ± 0.11	0.77
Isoleucine	1.89 ± 0.13	2.00 ± 0.14	0.37
Leucine	4.26 ± 0.06	4.47 ± 0.13	0.06
Tyrosine	1.83 ± 0.07	1.97 ± 0.13	0.19
Phenylalanine	2.35 ± 0.06	2.43 ± 0.09	0.28
Lysine	4.08 ± 0.14	4.21 ± 0.11	0.26
Histidine	1.35 ± 0.25	1.24 ± 0.05	0.40
Arginine	3.93 ± 0.06	3.97 ± 0.21	0.75
Proline	3.19 ± 0.30	3.28 ± 0.10	0.67
Total acid hydrolyzed AA	50.97 ± 0.74	53.33 ± 1.49	0.07

*CON, control group; LEU, leucine group. Values are presented as means ± Standard deviation (n = 15)*.

The contents of free Leu and threonine in semen of LEU was higher than CON (*P* < 0.05), while the contents of other FAA were not different to CON (*P* > 0.05). The contents of threonine, valine, methionine, isoleucine, phenylalanine, lysine, and alanine in boar sperm of LEU was not different to CON (*P* > 0.05), and the content of Leu showed an increasing trend, compared to CON (*P* = 0.05) (Table 9).

**Table 9:** Effect of leucine supplementation on FAA in boar seminal plasma (μmol/L) and AA of boar sperm (%).

**Items**	**CON**	**LEU**	***P-value***
Seminal plasma (μmol/L)
Threonine	80.40 ± 14.12	111.30 ± 35.41	0.04
Alanine	123.50 ± 37.44	133.00 ± 30.61	0.59
Valine	60.89 ± 14.33	51.25 ± 18.17	0.27
Methionine	3.90 ± 0.32	4.38 ± 1.12	0.36
Cysteine	11.17 ± 4.75	12.76 ± 3.26	0.47
Isoleucine	17.01 ± 6.34	20.71 ± 7.53	0.34
Leucine	29.24 ± 8.70	41.42 ± 11.11	0.04
Lysine	4.42 ± 1.54	5.89 ± 1.59	0.21
Arginine	6.59 ± 1.62	4.99 ± 1.88	0.12
Phenylalanine	27.58 ± 9.75	33.50 ± 17.89	0.43
Sperm (%)
Aspartic acid	4.23 ± 0.05	4.27 ± 0.03	0.40
Threonine	2.63 ± 0.10	2.65 ± 0.03	0.77
Serine	3.29 ± 0.10	3.24 ± 0.04	0.45
Glutamate	6.04 ± 0.23	6.28 ± 0.09	0.16
Glycine	1.98 ± 0.12	2.07 ± 0.02	0.30
Alanine	1.81 ± 0.19	1.93 ± 0.09	0.38
Cysteine	1.83 ± 0.12	1.97 ± 0.05	0.15
Valine	2.22 ± 0.13	2.36 ± 0.04	0.15
Methionine	0.75 ± 0.06	0.81 ± 0.02	0.17
Isoleucine	1.99 ± 0.10	2.09 ± 0.10	0.32
Leucine	3.43 ± 0.18	3.76 ± 0.10	0.05
Tyrosine	2.65 ± 0.13	2.73 ± 0.19	0.57
Phenylalanine	1.98 ± 0.12	2.04 ± 0.09	0.50
Lysine	3.19 ± 0.11	3.32 ± 0.08	0.17
Histidine	1.20 ± 0.04	1.20 ± 0.03	0.87
Arginine	7.60 ± 0.13	7.63 ± 0.55	0.95
Proline	3.14 ± 0.11	3.30 ± 0.09	0.11
Total acid hydrolyzed AA	49.97 ± 1.69	51.62 ± 0.53	0.18

*CON, control group; LEU, leucine group. Values are presented as means ± SD (n = 15)*.

### Testicular gene expressions

**Figure 3:**
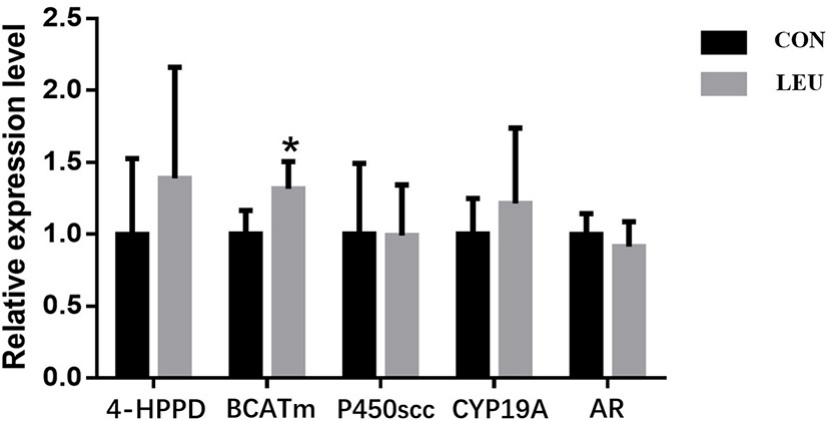
Effects of dietary leucine supplementation on leucine metabolism and steroid-related gene expression in the testis of boars. CON, control group; LEU, leucine group. Values are means, with their standard errors represented by vertical bars (*n* = 15), * means *P* < 0.05. 4-HPPD, 4-Hydroxyphenylpyruvate Dioxygenase; BCATm, branched-chain AA transaminase; P450scc, cytochrome P450 cholesterol side chain cleavage; CYP19A, aromatase; AR, androgen receptor.

The expression of Leu metabolism gene BCATm in testis of LEU was higher than that in CON (*P* < 0.05), while the expression of 4-HPPD was not different from that in CON (*P* > 0.05). Hormone synthesis related genes, such as CYP19A, P450scc and AR, were not different from that in CON (*P* > 0.05) (Figure 3).

**Figure 4:**
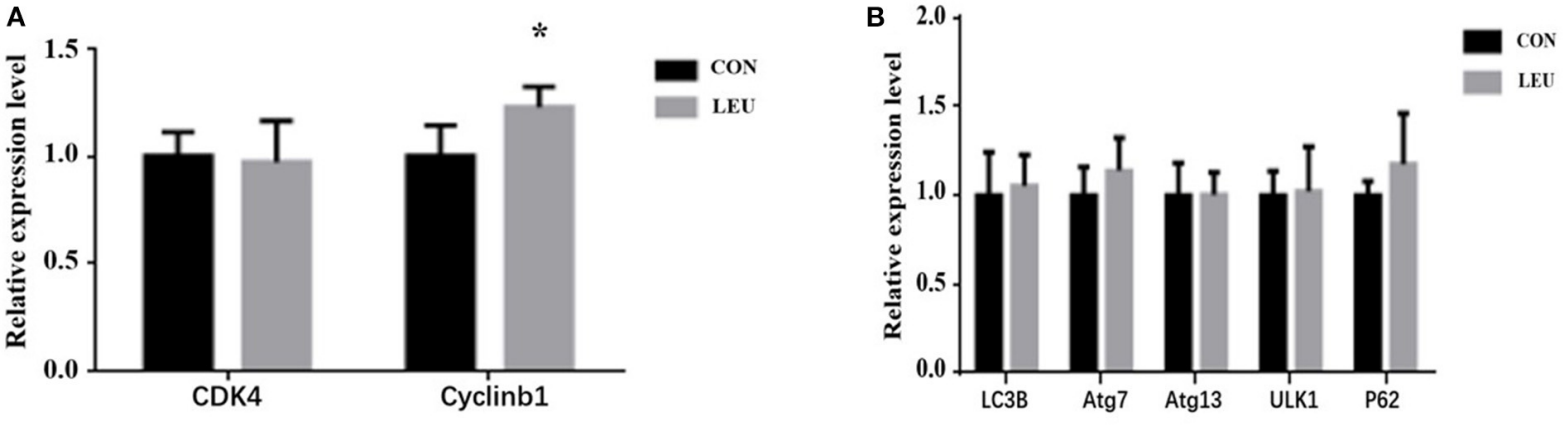
Effects of dietary leucine supplementation on gene expression related to cell proliferation **(A)** and autophagy **(B)** in the testis of boars. CON, control group; LEU, leucine group. Values are means, with their standard errors represented by vertical bars (*n* = 15), * means *P* < 0.05. CDK4, Cell Cycle Dependent Kinase 4; cyclinb1, cell cycle protein; LC3B, microtubule-associated protein 1 light chain 3b; Atg, autophagy-related gene; ULK1, Unc-like kinase 1; P62, autophagy-related protein 62.

The expression of Cyclinb1, a gene related to cell proliferation, in LEU was higher than that in CON (*P* < 0.05), while the expression of CDK4, another gene related to cell proliferation, was not different from that in CON (*P* > 0.05). The expression levels of genes related to autophagy, Atg13, P62, Atg7, and ULK1 in the testes of LEU were not different from those in CON (*P* > 0.05) (Figure 4).

**Figure 5:**
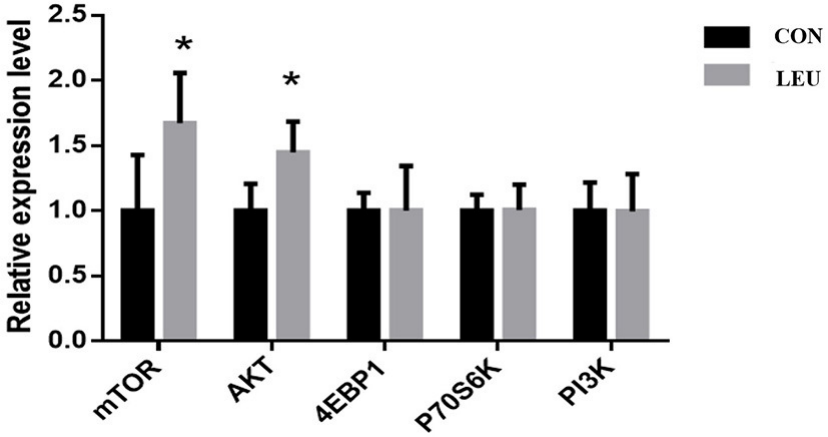
Effects of dietary leucine supplementation on expression of mTOR signaling pathway-related genes in the testis of boars. CON, control group; LEU, leucine group. Values are means, with their standard errors represented by vertical bars (*n* = 15), * means *P* < 0.05. mTOR, mammalian target of rapamycin; AKT, protein kinase B; 4EBP1, 4E-binding protein 1, Eukaryotic translation initiation factor 4 binding protein; P70S6K, p70 ribosomal protein S6 kinase; PI3K, creatine inositol 3 kinase.

The relative expression of AKT and mTOR genes in LEU was higher than that in CON (*P* < 0.05), while the relative expression of PI3K, 4EBP1, and P70S6K was not different from that in CON (*P* > 0.05) (Figure 5).

### Protein expression of MTOR, p-MTOR, and P70S6K1 in testis

**Figure 6:**
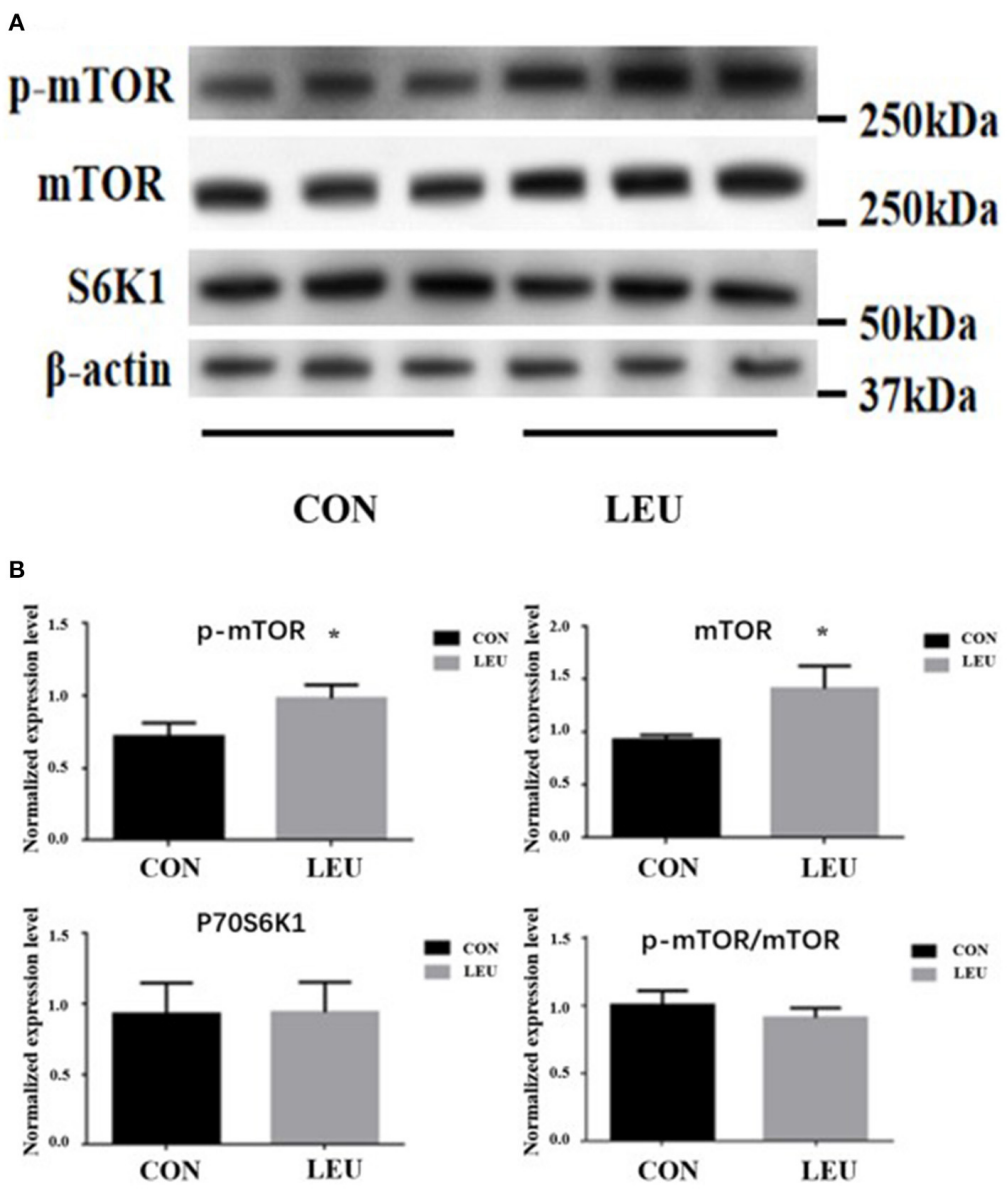
**(A,B)** Effects of dietary leucine supplementation on protein expression of mTOR signaling pathway in the testis of boars. CON, control group; LEU, leucine group. Values are means with their standard errors represented by vertical bars (*n* = 15). The * symbol indicates the values of *P* < 0.05.

The expression levels of p-mTOR and mTOR proteins in the testes of 150-days-old boars in LEU were increased compared to CON (*P* < 0.05). There was no difference in P70S6K1 protein, and the ratio of p-mTOR/mTOR protein compared to CON (*P* > 0.05) (Figure 6).

## Discussion

The development of boar testicles is an important indicator of reproductive performance. Cheng et al. found that the larger the testes of the boar were, the stronger their spermatogenesis was. The testicular development can be reflected by testicular volume and weight (26). Wei et al. found that dietary supplementation with L-arginine could increase testicular volume and weight. Similarly, in the current experiment, Leu increased testicular volume and weight (27). Testes are mainly composed of Sertoli cells, spermatogonia and Leydig cells. Kolahian et al. found that supplementation with Leu-containing water for 4 weeks in 8-week-old male, Wistar rats significantly increased the number of the Sertoli cells, and spermatogonia (5). Other studies have pointed out that an improvement in the structure of the convoluted seminiferous tubules was caused by increasing the number of cells (28). In this study, the number of Sertoli cells in the convoluted seminiferous tubules of the testes tended to increase after Leu supplementation. Studies have shown that the increase in Sertoli cells can provide a variety of growth factors for spermatogenic cells, which is conducive to their differentiation, and plays an important role in initiating pubertal development and maintaining spermatogenesis (28). Meanwhile, Leu supplementation in boar diet up-regulated the expression of Cyclinb1. Studies have shown that Cyclinb1 is crucial for mammalian reproduction and development. The expression of Cyclinb1 can be used as an indicator of the proliferation and differentiation ability of spermatogonia. Therefore, it can be speculated that the effect of Leu on testicular development is closely related to Sertoli cells proliferation.

The results of the current experiment also showed that dietary supplementation with 1.2% Leu could improve the semen quality of the boars. The semen quality of boars is an important indicator of reproductive performance. Kolahian et al. found that supplementation with Leu-containing water for 4 weeks in 8-weeks-old, male Wistar rats with diabetes significantly increased the total epididymal sperm count and motility (5). Zhang et al. found that VCL, VSL, and VAP of zebrafish sperm were significantly increased by intraperitoneal injection of Leu solution (4). Our results concur Leu increased the content of Leu in blood plasma and seminal plasma, and also tended to increase in testis and sperm, suggesting that Leu might absorbed into the blood and transferred to the testes and semen to improve semen quality. Dadoune et al. injected intra-venously and into the testes of male mice with 3H labeled AA and found that the radioactivity of AA was highest in Leydig cells, and type B spermatogonia, was lowest in sperm cells, and, among them, Leu showed the highest radioactivity (29). Free Leu in plasma can therefore effectively be used by the testes. Dong et al. found that dietary supplementation of AA could significantly affect the composition of FAA in boar sperm, thus affecting sperm motility (30). The current results showed that the expression of the Leu-metabolism gene BCATm in boar testes was significantly higher in the Leu group. Wiltafsky et al. found that BCATm expression was specific in pig tissues (31). BCATm can catalyze the transamination of branched-chain AA into branched-chain α-keto acids. This further confirms that Leu can be directly utilized by testicular tissue and can improve semen quality by affecting Leu metabolism.

Leu directly regulates the mTORC1 signaling pathway, largely independent of other AA (32). The mTORC1 can be regulated through the PI3K-AKT-mTOR signaling pathway (33). In recent years, more and more studies had shown that the mTOR signaling pathway plays an important role in the internal structure of testes, and the proliferation and differentiation of related cells (34). Our previous study showed that the key protein related to the promotion of testicular development after AA supplementation was mainly related to the mTORC1 signaling pathway (27). Meanwhile, Feng et al. found that activation of the AKT/mTOR signaling pathway can promote the cell cycle process of spermatogonia (35). Busada et al. found that the AKT/mTOR signaling pathway may be negatively regulated with c-kit to affect spermatogenesis (36). In this research, the expression of AKT and mTOR mRNA and p-mTOR and mTOR proteins were upregulated by Leu supplementation, which was similar to Yin et al. (2). Zhang et al. found that Leu enhanced the sperm motility of zebrafish by activating the PI3K-AKT-mTOR signaling pathway, which is also confirmed in our research (4). This further suggests that Leu may affect semen quality of boars by activating the mTOR signaling pathway (37).

## Conclusion

Dietary supplementation with 1.2% Leu from weaning to sexual maturity of boars increased the absorption and utilization of Leu in testicular tissue, promoted testicular development, and improved semen quality. Furthermore, the mTOR pathways were involved in the regulation of testicular development and spermatogenesis by Leu. These data offer the reproductive effect of orally administered Leu, which could provide essential guidance for the reasonable supplementation level and efficacy to male reproduction for future research.

## Data availability statement

The datasets presented in this study can be found in online repositories. The names of the repository/repositories and accession number(s) can be found in the article/supplementary material.

## Ethics statement

The animal study was reviewed and approved by Sichuan Agricultural University.

## Author contributions

YL takes responsibility for the integrity of data. YL, JiayL, KW, and DW designed the study. YL, JiayL, KW, BF, YZ, JianL, and DW conducted the study. YL, JiayL, KW, ZF, LC, SX, and DW analyzed the data. YL, JiayL, and KW wrote the manuscript. All authors have read and agreed to the published version of the manuscript.

## Funding

This research was funded by National 13th Five-Year Plan Key R&D Projects (2018YFD0501002) and the National Natural Science Foundation of China (No. 31702128).

## Conflict of interest

The authors declare that the research was conducted in the absence of any commercial or financial relationships that could be construed as a potential conflict of interest.

## Publisher's note

All claims expressed in this article are solely those of the authors and do not necessarily represent those of their affiliated organizations, or those of the publisher, the editors and the reviewers. Any product that may be evaluated in this article, or claim that may be made by its manufacturer, is not guaranteed or endorsed by the publisher.

## References

[1] Zhang S Qiao S Ren M Zeng X Ma X Wu Z. Supplementation with branched-chain amino acids to a low-protein diet regulates intestinal expression of amino acid and peptide transporters in weanling pigs. Amino Acids. (2013) 45:1191–205. 10.1007/s00726-013-1577-y 23990159

[2] Yin Y Yao K Liu Z Gong M Ruan Z Deng D. Supplementing L-leucine to a low-protein diet increases tissue protein synthesis in weanling pigs. Amino Acids. (2010) 39:1477–86. 10.1007/s00726-010-0612-5 20473536

[3] Columbus DA Steinhoff-Wagner J Suryawan A Nguyen HV Hernandez-Garcia A Fiorotto ML. Impact of prolonged leucine supplementation on protein synthesis and lean growth in neonatal pigs. Am J Physiol-endoc M. (2015) 309:E601–10. 10.1152/ajpendo.00089.2015 26374843 PMC4572453

[4] Zhang J Zhang X Liu Y Su Z Dawar FU Dan H. Leucine mediates autophagosome-lysosome fusion and improves sperm motility by activating the PI3K/Akt pathway. Oncotarget. (2017) 8:111807–18. 10.18632/oncotarget.22910 29340093 PMC5762361

[5] Kolahian S Sadri H Larijani A Hamidian G Davasaz A. Supplementation of diabetic rats with leucine, zinc, and chromium: effects on function and histological structure of testes. Int J Vitam Nutr Res. (2015) 85:311–21. 10.1024/0300-9831/a000244 27377195

[6] Zhang H Liu Y Zhou L Xu S Ye C Tian H. Metabonomic Insights into the Sperm Activation Mechanisms in Ricefield Eel (Monopterus albus). Genes (Basel). (2020) 11:1259. 10.3390/genes11111259 33114541 PMC7692440

[7] Ren B Cheng X Wu D Xu S Che L Fang Z. Effect of different amino acid patterns on semen quality of boars fed with low-protein diets. Anim Reprod Sci. (2015) 161:96–103. 10.1016/j.anireprosci.2015.08.010 26364704

[8] Gerlinger-Romero F Guimarães-Ferreira L Giannocco G Nunes MT. Chronic supplementation of beta-hydroxy-beta methylbutyrate (HMβ) increases the activity of the GH/IGF-I axis and induces hyperinsulinemia in rats. Growth Horm IGF Res. (2011) 21:57–62. 10.1016/j.ghir.2010.12.006 21237681

[9] Xu G Rothenberg PL. Insulin receptor signaling in the β-Cell influences insulin gene expression and insulin content: evidence for autocrine β-Cell regulation. Diabetes. (1998) 47:1243–52. 10.2337/diabetes.47.8.1243 9703324

[10] Neirijnck Y Calvel P Kilcoyne KR Kühne F Stévant I Griffeth RJ. Insulin and IGF1 receptors are essential for the development and steroidogenic function of adult Leydig cells. FASEB J. (2018) 32:3321–35. 10.1096/fj.201700769RR 29401624

[11] Hu G Lin H Chen G Chen B Lian Q Hardy DO. Deletion of the Igf1 gene: suppressive effects on adult Leydig cell development. J Androl. (2010) 31:379–87. 10.2164/jandrol.109.008680 20203337 PMC4103413

[12] Laufs U Banach M Mancini GBJ Gaudet D Bloedon LT Sterling LR. Efficacy and safety of bempedoic acid in patients with hypercholesterolemia and statin intolerance. J Am Heart Assoc. (2019) 8:e011662. 10.1161/JAHA.118.011662 30922146 PMC6509724

[13] Schade DS Shey L Eaton RP. Cholesterol review: a metabolically important molecule. Endocr Pract. (2020) 26:1514–23. 10.4158/EP-2020-0347 33471744

[14] Lynch CJ Gern B Lloyd C Hutson SM Eicher R Vary TC. Leucine in food mediates some of the postprandial rise in plasma leptin concentrations. Am J Physiol Endocrinol Metab. (2006) 291:E621–30. 10.1152/ajpendo.00462.2005 16638821

[15] Yuan XW Han SF. Zhang JW, Xu JY, Qin LQ. Leucine supplementation improves leptin sensitivity in high-fat diet fed rats. Food Nutr Res. (2015) 59:27373. 10.3402/fnr.v59.27373 26115673 PMC4482813

[16] Mao X Zeng X Wang J Qiao S. Leucine promotes leptin receptor expression in mouse C2C12 myotubes through the mTOR pathway. Mol Biol Rep. (2011) 38:3201–6. 10.1007/s11033-010-9992-6 20151325

[17] Hoffmann A Manjowk GM Wagner IV Klöting N Ebert T Jessnitzer B. Leptin within the subphysiological to physiological range dose dependently improves male reproductive function in an obesity mouse model. Endocrinology. (2016) 157:2461–8. 10.1210/en.2015-1966 27105383

[18] NRC. Nutrient Requirements of Swine. 11th ed. Washington, DC: The National Academies Press (2012). 10.17226/13298

[19] Lambert B. The frequency of mumps and of mumps orchitis and the consequences for sexuality and fertility. Acta Genet Stat Med. (1951) 2:1–166. 15444009

[20] Louis GF Lewis AJ Weldon WC Miller PS Kittok RJ Stroup WW. The effect of protein intake on boar libido, semen characteristics, and plasma hormone concentrations. J Anim Sci. (1994) 72:2038–50. 10.2527/1994.7282038x 7982833

[21] Ding H Luo Y Liu M Huang J Xu D. Histological and transcriptome analyses of testes from Duroc and Meishan boars. Sci Rep. (2016) 6:20758. 10.1038/srep20758 26865000 PMC4749976

[22] Daniel MK Mark JE Allen FH. The effect of lutalyse on the training of sexually inexperienced boars for semen collection. Theriogenology. (2002) 58:1039–45. 10.1016/S0093-691X(02)00938-X 12212885

[23] Sun L Fan X Zeng Y Wang L Zhu Z Li R. Resveratrol protects boar sperm in vitro via its antioxidant capacity. Zygote. (2020) 2:1–8. 10.1017/S0967199420000271 32482196

[24] Hu L Liu Y Yan C Peng X Xu Q Xuan Y. Postnatal nutritional restriction affects growth and immune function of piglets with intra-uterine growth restriction. Br J Nutr. (2015) 114:53–62. 10.1017/S0007114515001579 26059215

[25] Che L Xu Q Wu C Luo Y Huang X Zhang B. Effects of dietary live yeast supplementation on growth performance, diarrhoea severity, intestinal permeability and immunological parameters of weaned piglets challenged with enterotoxigenic Escherichia coli K88. Br J Nutr. (2017) 118:949–58. 10.1017/S0007114517003051 29166952

[26] Lin Y Cheng X Mao J Wu D Ren B Xu S. Effects of different dietary n-6/n-3 polyunsaturated fatty acid ratios on boar reproduction. Lipids Health Dis. (2016) 15:31. 10.1186/s12944-016-0193-8 26884231 PMC4756391

[27] Lin Y Wei D Wang K Wu D Zhang J Che L. Proteomic analysis reveals key proteins involved in arginine promotion of testicular development in boars. Theriogenology. (2020) 154:181–9. 10.1016/j.theriogenology.2020.05.027 32622198

[28] Cupp AS Skinner MK. Expression, action, and regulation of transforming growth factor alpha and epidermal growth factor receptor during embryonic and perinatal rat testis development. J Androl. (2001) 22:1019–29. 10.1002/j.1939-4640.2001.tb03443.x 11700850

[29] Dadoune JP Fain-Maurel MA Alfonsi MF Katsanis G. In vivo and in vitro radioautographic investigation of amino acid incorporation into male germ cells. Biol Reprod. (1981) 24:153–62. 10.1095/biolreprod24.1.153 6781546

[30] Dong H Wu D Xu S Li Q Fang Z Che L. Effect of dietary supplementation with amino acids on boar sperm quality and fertility. Anim Reprod Sci. (2016) 172:182–9. 10.1016/j.anireprosci.2016.08.003 27509874

[31] Wiltafsky MK Pfaffl MW Roth FX. The effects of branched-chain amino acid interactions on growth performance, blood metabolites, enzyme kinetics and transcriptomics in weaned pigs. Br J Nutr. (2010) 103:964–76. 10.1017/S0007114509992212 20196890

[32] Rudar M Zhu C De Lange CF. Dietary leucine supplementation decreases whole-body protein turnover before, but not during, immune system stimulation in pigs. J Nutr. (2017) 147:45–51. 10.3945/jn.116.236893 27798336

[33] Frey JW Jacobs BL Goodman CA Hornberger TA. A role for raptor phosphorylation in the mechanical activation of mTOR signaling. Cell Signal. (2014) 26:313–22. 10.1016/j.cellsig.2013.11.009 24239769 PMC3917221

[34] Deng C Lv M Luo B Zhao S Mo Z Xie Y. The role of the PI3K/AKT/mTOR signalling pathway in male reproduction. Curr Mol Med. (2021) 21:539–48. 10.2174/1566524020666201203164910 33272176

[35] Feng L Ravindranath N Dym M. Stem cell factor/c-kit up-regulates cyclin D3 and promotes cell cycle progression via the phosphoinositide 3-kinase/p70 S6 kinase pathway in spermatogonia. J Biol Chem. (2000) 275:25572–6. 10.1074/jbc.M002218200 10849422

[36] Busada JT Chappell VA Niedenberger BA Kaye EP Keiper BD Hogarth CA. Retinoic acid regulates kit translation during spermatogonial differentiation in the mouse. Dev Biol. (2015) 397:140–9. 10.1016/j.ydbio.2014.10.020 25446031 PMC4268412

[37] Chantranupong L Scaria SM Saxton RA Gygi MP Shen K Wyant GA. The CASTOR proteins are arginine sensors for the mTORC1 pathway. Cell. (2016) 165:153–4. 10.1016/j.cell.2016.02.035 26972053 PMC4808398

