# Supplementary material for: Effects of dietary L-leucine supplementation on testicular development and semen quality in boars
Source: Front Vet Sci. 2022 Jul 15;9:904653. doi: 10.3389/fvets.2022.904653 (PMC9334790; doi:10.3389/fvets.2022.904653)
Supplement: Supplementary file 1 [file Table_1.DOCX]

**TABLE 1** Nutrient levels of the basal diets

| **Item** | **7-11kg** | **11-25kg** | **25-50kg** | **50-100kg** | **100-130kg** | **130kg-** |
| --- | --- | --- | --- | --- | --- | --- |
| Digestible energy, Mcal/kg | 3.58 | 3.51 | 3.42 | 3.41 | 3.39 | 3.25 |
| Crude protein, % | 20.46 | 19.49 | 17.50 | 16.64 | 15.12 | 15.19 |
| Ca, % | 0.80 | 0.70 | 0.70 | 0.70 | 0.70 | 0.70 |
| Available phosphorus, % | 0.42 | 0.33 | 0.34 | 0.34 | 0.34 | 0.32 |
| Total lysine, % | 1.18 | 1.12 | 1.11 | 1.02 | 0.80 | 0.77 |
| Total methionine, % | 0.30 | 0.26 | 0.38 | 0.28 | 0.20 | 0.11 |
| Total threonine, % | 0.86 | 0.69 | 0.73 | 0.66 | 0.60 | 0.57 |
| Total valine, % | 0.83 | 0.75 | 0.72 | 0.69 | 0.67 | 0.58 |
| Total isoleucine, % | 0.70 | 0.64 | 0.62 | 0.62 | 0.61 | 0.59 |
| Total leucine, % | 1.50 | 1.35 | 1.45 | 1.39 | 1.75 | 1.61 |
