# Supplementary material for: Effects of dietary L-leucine supplementation on testicular development and semen quality in boars
Source: Front Vet Sci. 2022 Jul 15;9:904653. doi: 10.3389/fvets.2022.904653 (PMC9334790; doi:10.3389/fvets.2022.904653)
Supplement: Supplementary file 2 [file Table_2.DOCX]

**TABLE S2** Primer sequences used for quantitative real-time PCR

| **Genes** | **Primer sequences（5’-3’）** | **Gene bank No.** |
| --- | --- | --- |
| 4-HPPD -F | GTCTTCTCCTCTGCCCTCAA | XM_021072173.1 |
| 4-HPPD -R | GCAAATTCACCTTCCCAAAC |  |
| BCATm -F | GCCTGAAGGCGTACAAAGG | XM_005664688.3 |
| BCATm -R | GATGCACTCCAGCAACTCG |  |
| 4EBP1-R | GGTTCTGGCTGGCATCTGT | NM_004095.3 |
| 4EBP1-F | CCGGAAGTTCCTAATGGAGTGT |  |
| P70S6K-R | TTGGAAGTGGTGCAGAAGCTT | XM_003131671.4 |
| P70S6K-F | GGAAACAAGTGGAATAGAGCAGATG |  |
| mTOR-R | CCTTTCGAGATGGCAATGGA | XM_003127584.6 |
| mTOR-F | TTGTTGCCCCCTATTGTGAAG |  |
| PI3K-R | AACCACGCTTCAGCAGAAAT | XM_021102206.1 |
| PI3K-F | CCAGAAACTGGAGAGCTTGG |  |
| P450SCC-F | TCGGCAACTTGGAATCTGTT | XM_021098320.1 |
| P450SCC-R | AATGCTGGTGATAGGCAACC |  |
| CYP19A-F | TGGAGTGCATCGGCATGTAT | NM_214430.1 |
| CYP19A-R | GTGATGGAATCGGCACAGAC |  |
| AKT-F | GAGGTCATGGAGCACAGGTT | NM_001256779.1 |
| AKT-R | CGTCAAAGTACCGAGTGTCG |  |
| AR-F | ACGAGAAACAGCAGCCTTCA | NM_214314.2 |
| AR-R | AGCTGCTTAAACCCGGGAAA |  |
| Cyclinb1-F | TGGTGAATGGACACCAACTC | NM_001170768.1 |
| Cyclinb1-R | TGCTTTGTAAGCCCTCGATT |  |
| CDK4-F | GCATCCCAATGTTGTCCG | NM_001123097.1 |
| CDK4-R | GGGGTGCCTTGTCCAGATA |  |
| LC3B-F | CCGAACCTTCGAACAGAGAG | NM_001190290.1 |
| LC3B-R | AGGCTTGGTTAGCATTGAGC |  |
| P62-F | GGGTTACACCAGCAGTCCA | XM_003123639.4 |
| P62-R | CCATTCAGAGTACATCCGTTT |  |
| Agt13-F | CAGCCTCTCCATAGATATTG | XM_005660951.3 |
| Agt13-R | TGCAGGGTTTCTACAAAG |  |
| ULK1-F | CGAGAAACATGACCTGGA | XM_021072525.1 |
| ULK1-R | GGATCTTGATTTCCTTCCC |  |
| Agt7-F | AGATTGCCTGGTGGGTGGT | XM_021068402.1 |
| Agt7-R | GGGTGATGCTGGAGGAGTTG |  |
| β-actin-F | TCTGGCACCACACCTTCT | U07786.1 |
| β-actin-R | TGATCTGGGTCATCTTCTCAC |  |
